# Nuclear NPM-ALK Protects Myc from Proteasomal Degradation and Contributes to Its High Expression in Cancer Stem-Like Cells in ALK-Positive Anaplastic Large Cell Lymphoma

**DOI:** 10.3390/ijms241814337

**Published:** 2023-09-20

**Authors:** Chuquan Shang, Justine Lai, Moinul Haque, Will Chen, Peng Wang, Raymond Lai

**Affiliations:** 1 Department of Laboratory Medicine and Pathology, University of Alberta, Edmonton, AB T6G 2R3, Canada; chuquan@ualberta.ca (C.S.); moinul@ualberta.ca (M.H.); will.chen@ualberta.ca (W.C.); 2 Department of Medicine, Division of Hematology, University of Alberta, Edmonton, AB T6G 2R3, Canada; jlai@ualberta.ca (J.L.); pw2@ualberta.ca (P.W.); 3 Department of Pathology, Yale School of Medicine, New Haven, CT 06510, USA; 4 Department of Oncology, Cross Cancer Institute, Edmonton, AB T6G 1Z2, Canada

**Keywords:** ALK-positive anaplastic lymphoma, Myc, cancer stemness, proteasomal degradation

## Abstract

In ALK-positive anaplastic large cell lymphoma (ALK+ALCL), a small subset of cancer stem-like (or RR) cells characterized by high Myc expression have been identified. We hypothesize that NPM-ALK contributes to their high Myc expression. While transfection of *NPM-ALK* into HEK293 cells effectively increased Myc by inhibiting its proteosomal degradation (PD-Myc), this effect was dramatically attenuated when the full-length NPM1 (FL-NPM1) was downregulated using shRNA, highlighting the importance of the NPM-ALK:FL-ALK heterodimers in this context. Consistent with this concept, immunoprecipitation experiments showed that the heterodimers are abundant only in RR cells, in which the half-life of Myc is substantially longer than the bulk cells. Fbw7γ, a key player in PD-Myc, is sequestered by the heterodimers in RR cells, and this finding correlates with a Myc phosphorylation pattern indicative of ineffective PD-Myc. Using confocal microscopy and immunofluorescence staining, we found that the fusion signal between ALK and FL-NPM1, characteristic of the heterodimers, correlates with the Myc level in ALK+ALCL cells from cell lines and patient samples. To conclude, our findings have revealed a novel oncogenic function of NPM-ALK in the nucleus. Specifically, the NPM-ALK:FL-NPM1 heterodimers increase cancer stemness by blocking PD-Myc and promoting Myc accumulation in the cancer stem-like cell subset.

## 1. Introduction

ALK (anaplastic lymphoma kinase)-positive anaplastic large cell lymphoma, or ALK+ALCL, is a type of aggressive non-Hodgkin lymphoma of the mature T-cell immunophenotype recognized in the World Health Organization Classification Scheme [1]. For 70–80% of these neoplasms, the binding partner of ALK is nucleophosmin (NPM1); in the remaining subset, ALK has been found to fuse with various partners, thereby creating many different fusion ALK proteins, including RNF213-ALK (or ALO17-ALK), TFG-ALK, MSN-ALK, TPM3-ALK, TPM4-ALK ATIC-ALK, MYH9-ALK, and CLTC-ALK [2]. While the pathogenesis of NPM-ALK has been extensively studied, that of the less frequently encountered ALK fusion proteins is incompletely understood, although one study has shown that ATIC-ALK carries less transforming ability than NPM-ALK [2]. Regarding NPM-ALK, this fusion protein results from the reciprocal translocation of *2p23* (the *ALK* gene) and *5q35* (the *NPM1* gene), and it has been shown to be a constitutively active tyrosine kinase [3]. The truncated N-portion of NPM1 is responsible for the homodimerization of the fusion proteins, a configuration that triggers the autophosphorylation of multiple residues on NPM-ALK and the constitutive activation of the tyrosine kinase embodied in the truncated C-portion of ALK [4]. NPM-ALK mediates much of its oncogenic effects by binding to and activating a host of signaling proteins, thereby deregulating multiple cellular pathways that normally exert control over cell survival [5], proliferation [5,6], metabolism [7,8], the cytoskeleton [9,10,11], immune evasion [12,13,14], and maintenance of the genome integrity [15,16,17]. Accordingly, deregulations of these cellular pathways generate widespread oncogenic effects. In a review published in 2017, NPM-ALK is believed to contribute to all but 1 of the 10 hallmarks of cancer cells [18]. These oncogenic events occur primarily in the cytoplasm of ALK+ALCL cells, because evidence of autophosphorylation of NPM-ALK, a marker of homodimerization of NPM-ALK, is negligible or not detectable in the nuclei of ALK+ALCL cells [19]. It is believed that nuclear NPM-ALK proteins exist almost exclusively as heterodimers consisting of NPM-ALK and full-length NPM1 (FL-NPM1), and this concept is based on two observations. Firstly, as mentioned, subcellular fractionation/Western blot and immunohistochemical studies have shown that nuclear NPM-ALK proteins lack significant autophosphorylation, a marker of homodimerization [19]. Second, while the FL-NPM1 protein carries a nucleus/nucleolus localization signal, the NPM1 represented in NPM-ALK does not carry this signal due to the truncation [19]. Thus, it is widely believed that the formation of the NPM-ALK:FL-NPM1 heterodimers (hereafter heterodimers) is required for the nuclear localization of NPM-ALK. Nonetheless, to our knowledge, there has been no direct evidence showing that nuclear NPM-ALK proteins persist as heterodimers. The biological functions of nuclear NPM-ALK are also largely unknown, although we have recently provided evidence that nuclear NPM-ALK can promote the transcriptional activity of FoxM1 and its oncogenic effects in ALK+ALCL cells [20].

More recent research has shed light on the existence of tumor heterogeneity and its biological/clinical significance [21,22,23]. Cancer stem cells and cancer stem-like (CSL) cells have been postulated to be key contributors to cancer relapses [24,25]. The identification and characterization of cancer stem cells and CSL cells is an active area of cancer research. Using a commercially available SRR2 reporter, which was designed to detect the transcriptional activity of the embryonic stem cell protein Sox2, we had previously identified the existence of a small subset of CSL cells in ALK+ALCL, labeled as Reporter Responsive or RR cells [26]. Compared to the Reporter Unresponsive or RU cells, RR cells display significantly higher chemoresistance and tumorigenicity. While Sox2 is highly expressed in both RU and RR cells, only the latter carry detectable Sox2 transcriptional activity, which contributes to their SRR2 reporter activity. In addition, we also found that the expression of Myc, another embryonic stem cell protein, is substantially higher in RR cells. Importantly, Myc directly contributes to the SRR2 reporter activity and CSL phenotype in RR cells. While Myc is one of the most implicated oncoproteins in human cancer [27,28,29,30], its pathogenetic roles in ALK+ALCL have not been well-defined, although a handful of previous studies have shown that Myc is a downstream target of NPM-ALK via STAT3 signaling [31,32]. Because Myc contributes to the CSL phenotype of RR cells, we believe that it is highly relevant to investigate the mechanism underlying the differential Myc protein levels between RU and RR cells.

The major objective of this study is to decipher the mechanism underlying the differential expression levels of Myc proteins between RU and RR cells. Our results have revealed the importance of the proteasome degradation of Myc (PD-Myc) in this context, as we found evidence that the high Myc protein expression in RR cells is attributed to the relative ineffectiveness of this pathway in these cells. Our data also led us to challenge the paradigm that nuclear NPM-ALK proteins exist predominantly as the heterodimers in ALK+ALCL cells because this appears to hold true only in RR cells, a minority of the total cell population. Nonetheless, while the heterodimers may not exist as ubiquitously in the nuclei of ALK+ALCL as previously expected, our results suggest these proteins play an important role in deregulating PD-Myc and allowing the accumulation of Myc in the CSL cell subset.

## 2. Results

### 2.1. NPM-ALK Upregulates Myc by Decreasing Its Proteasomal Degradation

**Figure 1:**
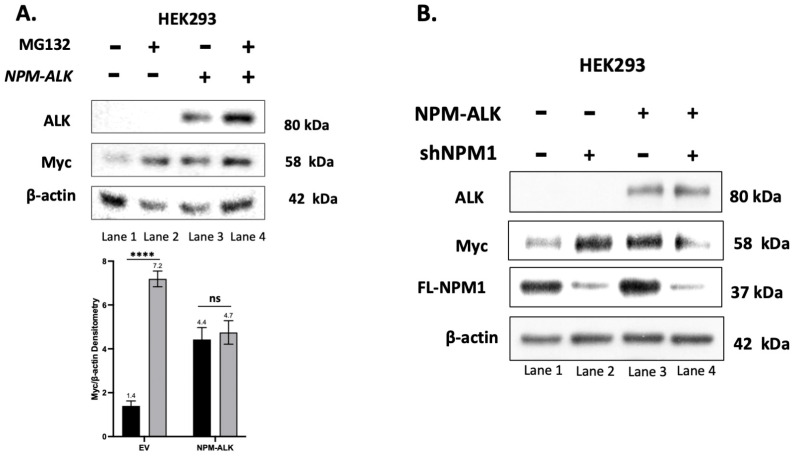
NPM-ALK promotes Myc accumulation in HEK293 cells. (**A**). Western blot studies showed a substantial increase in Myc proteins in HEK293 cells after *NPM-ALK* gene transfection (lane 1 vs. lane 3). In the absence of NPM-ALK, the addition of proteasome inhibitor MG132 (10 μM) for 2 h also increased the expression of Myc (lane 1 vs. lane 2), and this finding suggests that PD-Myc is relatively efficient in HEK293 cells. By contrast, in the presence of exogenous NPM-ALK, Myc did not increase appreciably (lane 3 vs. lane 4). Taken together, these results suggest that NPM-ALK increased the protein level of Myc by inhibiting PD-Myc. Numbers shown in the histogram represent band densitometry results. Triplicate experiments were performed and results from a representative experiment are shown. The statistical significance is assessed by using Student’s *t*-test. **** *p* < 0.0001 (**B**). Western blot studies showed that shRNA knockdown of FL-NPM1 resulted in a marked accumulation of Myc in HEK293 cells transduced with an empty vector (lane 1 vs. lane 2). In contrast, in the presence of exogenous NPM-ALK, shRNA knockdown of FL-NPM1 decreased Myc (lane 3 vs. lane 4). Of note, the NPM-ALK protein level was not affected by the knockdown of FL-NPM1. Triplicates were performed for this experiment and results from a representative experiment are shown.

Previous studies have suggested that NPM-ALK can upregulate the expression of Myc [31,32]. To further establish the link between NPM-ALK and Myc, we transfected *NPK-ALK* into HEK293 cells. As shown in Figure 1A (lane 1 vs. lane 3), the expression of NPM-ALK resulted in a substantial increase in Myc detectable by Western blots. Similar findings were observed by using immunofluorescence staining and confocal microscopy (Supplemental Figure S1). We then asked if this NPM-ALK-induced upregulation of Myc is related to the proteasomal degradation of Myc (PD-Myc). The dramatic upregulation of Myc upon the addition of the proteasome inhibitor MG132 (Figure 1A, lane 1 vs. lane 2) strongly suggests that the PD-Myc machinery is relatively effective in HEK293 cells in the absence of NPM-ALK. In comparison, the addition of MG132 to NPM-ALK-transfected HEK293 cells led to a minimal increase in Myc (lane 3 vs. lane 4), suggesting that NPM-ALK impairs PD-Myc and allows Myc to accumulate.

### 2.2. NPM-ALK-Mediated Inhibition of PD-Myc Is Dependent on Full-Length NPM1

As mentioned in the Introduction, it is widely believed that the NPM-ALK proteins found in the nuclei of ALK+ALCL cells exist predominantly as heterodimers (i.e., NPM-ALK:FL-NPM) [19]. In another line of studies, it has been published that FL-NPM1 can physically bind to Myc [33,34]. Taken together, we hypothesize that the heterodimers migrate to the nuclei and sequester/protect Myc from being degraded. 

To test this hypothesis, we examined the effects of the shRNA knockdown of FL-NPM1 in the absence/presence of NPM-ALK transfection in HEK293 cells. For this experiment, we employed shRNA species designed to target the C-terminal of the NPM1; because the truncated NPM1 portion of NPM-ALK does not carry the C-terminal, the expression of this fusion protein should not be affected. As shown in Figure 1B, in the absence of NPM-ALK transfection, the shRNA knockdown of FL-NPM1 effectively decreased its expression level at 37 kDa, and this change correlated with a substantial increase in the Myc protein level (lane 1 vs. lane 2). These results are consistent with the concept that FL-NPM1 is a key component of the PD-Myc machinery in several cell types [34,35,36,37]. In sharp contrast, the shRNA knockdown of FL-NPM1 decreased the Myc protein level in cells transfected with NPM-ALK (lane 3 vs. lane 4). These findings are consistent with our hypothetical model that, while FL-NPM1 is normally a key component of the PD-Myc machinery, it is utilized by NPM-ALK in the form of heterodimers to sequester Myc and protect it from being degraded.

### 2.3. The Efficiency of PD-Myc Is Different between RU and RR Cells

**Figure 2:**
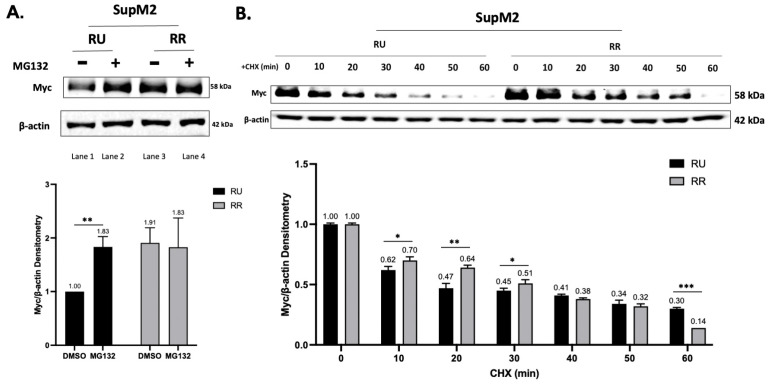
Myc degradation is inefficient in RR cells compared to RU cells. (**A**). Western blot studies showed that RU and RR cells derived from SupM2 were substantially different in their Myc protein level (lane 1 vs. lane 3). Furthermore, while treatment with MG132 (10 μM) for 2 h induced an appreciable upregulation of Myc in RU cells (lane 1 vs. lane 2), the Myc level did not change substantially in response to the same treatment in RR cells (lane 3 vs. lane 4). Band densitometry was calculated by ImageStudioLite software and histograms were made by Graphpad. Results were shown in the lower panel. (**B**). Results from the time course experiment highlighting the difference in the rate of Myc degradation in RU and RR cells are illustrated. A total of 10 μM of cycloheximide (CHX) was used in this experiment. Cells were collected every 10 min. Numbers shown in the histograms represent band densitometry results calculated by ImageStudioLite and data were shown as mean ± standard deviation (SD), *n* = 3, * *p* < 0.05, ** *p* < 0.01, *** *p* < 0.001, Student’s *t*-test. Band densitometry was normalized to RU or RR cells at 0 min, respectively. The half-life of RU cells was around 10–20 min, whereas that of RR cells was around 30–40 min. Three independent experiments were performed and results from a representative experiment are shown.

Our group has previously reported that ALK+ALCL cell lines consistently contain a small subset of CSL cells (labeled RR cells), which are phenotypically distinct from the bulk cells (labeled RU cells) [26]. Furthermore, Myc, which is expressed substantially higher in RR cells, is the key driver of the CSL phenotype [38,39,40]. As shown in Figure 2A, the RR cells expressed substantially higher Myc than the RU cells in the steady state (lane 1 vs. lane 3). These two cell subsets responded to MG132 very differently, with Myc being dramatically upregulated in the RU cells (lane 1 vs. lane 2) and being relatively unchanged in the RR cells (lane 3 vs. lane 4). These findings suggest that PD-Myc is relatively efficient in RU cells but not RR cells, which likely contributes to the relatively high Myc protein level in the RR cells. In further support of this concept, the results from our Myc half-life experiments showed that the Myc proteins lingered substantially longer in the RR cells after cycloheximide treatment than in the RU cells (Figure 2B).

### 2.4. FL-NPM1 Is Important in Inhibiting PD-Myc in RR but Not RU Cells

**Figure 3:**
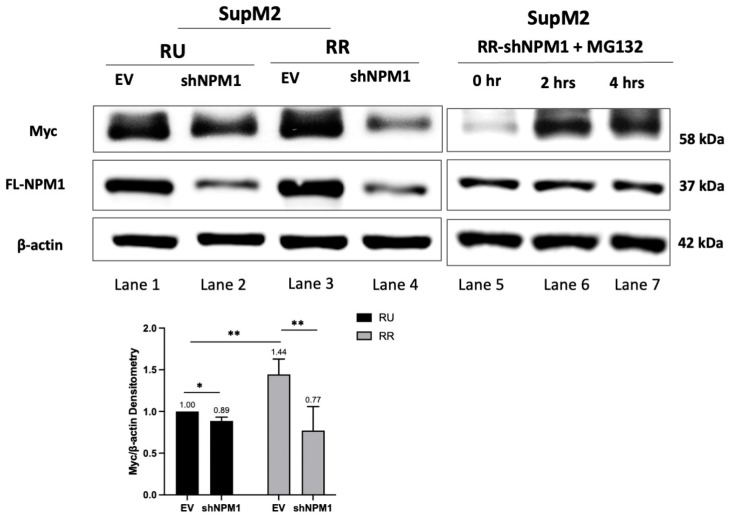
NPM1 is a protector of Myc due to the inefficient Myc degradation in RR but not the RU subset. Left panel: Western blot analysis of RU and RR cells derived from SupM2 before and after FL-NPM1 knockdown. The change in the Myc level was relatively small in RU cells (lane 1 vs. lane 2, band intensity 1.00 vs. 0.89, *p* > 0.05), probably due to the fact that PD-Myc is relatively efficient in these cells and the residual FL-NPM1 is sufficient to prevent the protein accumulation of Myc. In contrast, the same treatment led to a dramatic decrease in Myc in RR (lane 3 vs. lane 4, band intensity 1.44 vs. 0.77, *p* > 0.01), in keeping with our model that FL-NPM1-mediated PD-Myc is different between RU and RR cells. Right panel: After shRNA knockdown of FL-NPM1, RR cells were treated with 10 μM MG132, and cells were collected at 0, 2, and 4 h. The accumulation of Myc (lane 5 vs. lane 6 and 7) after MG132 treatment supports the concept that the Myc decrease due to the knockdown of FL-NPM1 in RR cells was via the inhibition of the PD-Myc pathway. Band densitometry was calculated by ImageStudioLite software and results were normalized to RU-EV group. Student’s *t*-test was performed using GraphPad and results were shown in the lower panel, * *p* < 0.05, ** *p* < 0.01. Triplicate experiments were performed and results from a representative experiment are illustrated.

If our hypothetical model is correct, we expect that the shRNA knockdown of FL-NPM1 in RR cells will substantially decrease Myc, in parallel with what we observed for HEK293 cells transfected with NPM-ALK. This turned out to be the case. While the shRNA knockdown of FL-NPM1 resulted in only a subtle change in Myc in the RU cells (Figure 3, left panel, lane 1 vs. lane 2), the same treatment led to a dramatic downregulation of Myc (Figure 3, left panel, lane 3 vs. lane 4). Because PD-Myc is relatively efficient in RU cells, it is likely that the residual FL-NPM1 (after shRNA treatment) was adequate to prevent Myc accumulation in these cells, and this concept correlates with our observation. To confirm if the decrease in Myc in the RR cells treated with FL-NPM1 knockdown is indeed due to a change in PD-Myc, we treated these cells with MG132. As shown in Figure 3B, the addition of MG132 to the RR cells in which FL-NPM1 had been knocked down resulted in a dramatic upregulation of Myc between 2 and 4 h (Figure 3, right panel, lane 5–7). Overall, our findings are consistent with our hypothetical model that FL-NPM1 as a component of the heterodimers upregulates Myc expression in RR cells by disrupting PD-Myc.

### 2.5. Inhibition of ALK Restores PD-Myc in RR Cells

**Figure 4:**
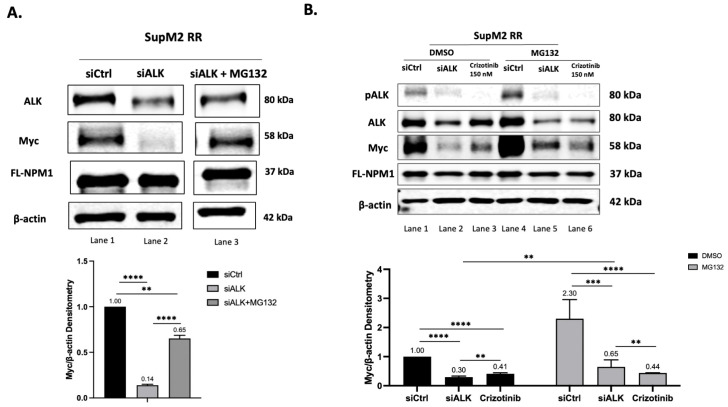
NPM-ALK contributes to the high Myc protein level in RR cells by inhibiting its proteasomal degradation. (**A**). Western blot studies were performed using lysates from RR cells (SupM2) treated with siRNA to knockdown ALK for 24 h. Myc expression decreased dramatically (lane 1 vs. lane 2, band intensity 1.00 vs. 0.30, *p* < 0.0001). The addition of 10 μM of MG132 to the cell culture for 2 h effectively restored the expression of Myc, suggesting that NPM-ALK promotes Myc accumulation by blocking PD-Myc. All bands shown were obtained on the same blot. (**B**). A similar experiment was performed, but cells treated with 150 nM of Crizotinib were included. While Crizotinib also decreased Myc expression, the use of MG132 failed to effectively restore its expression (lane 3 vs. lane 6, band intensity 0.41 vs. 0.44, *p* = 0.28). Band densitometry was calculated by ImageStudioLite software and results were normalized to RR-siCtrl with or without DMSO groups, respectively. Student’s *t*-test was performed using GraphPad and results were shown in the lower panel, ** *p* < 0.01, *** *p* < 0.001, **** *p* < 0.0001. Triplicate experiments were performed and results from a representative run are shown.

Because our hypothetical model hinges on the role of the heterodimers in disrupting PD-Myc, the suppression of the NPM-ALK expression, the other component of the NPM-ALK:FL-NPM1 dimer, is also expected to result in the restoration of PD-Myc and a decrease in the Myc protein level. The results shown in Figure 4 are consistent with this model. Firstly, as shown in Figure 4A, in the RR cells, the knockdown of ALK using siRNA dramatically decreased the Myc protein level (lane 1 vs. lane 2), and this effect was reversible upon the addition of MG132 (lane 3). As shown in Figure 4B, pharmacologic inhibition of the tyrosine activity of ALK using a low dose of Crizotinib decreased the Myc protein level (lane 1 vs. lane 3), although this effect was weaker than that of siRNA (lane 2 vs. lane 3). The Crizotinib-induced decrease of Myc could not be effectively restored by MG132 (lane 3 vs. lane 6), suggesting that the interference of PD-Myc by NPM-ALK requires the presence of the protein structure rather than the tyrosine kinase activity of NPM-ALK. This is in keeping with our model.

### 2.6. The NPM-ALK:FL-NPM1 Heterodimers Are Found Largely in RR Cells but Not RU Cells

**Figure 5:**
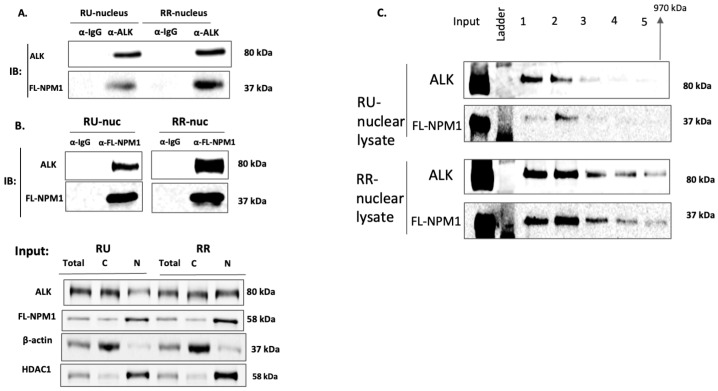
Nuclear NPM-ALK:FL-NPM1 heterodimers are more abundant in RR cells. (**A**,**B**). Immunoprecipitation experiments were performed using cell lysates from RU and RR cells (derived from SupM2) and antibodies against either ALK ((**A**). left top panel) or FL-NPM1 ((**B**). left middle panel). Results from both experiments support that the NPM-ALK:FL-NPM1 heterodimers are more abundant in RR cells. Protein inputs are shown in the left bottom panel. (**C**). Nuclear lysates from RU and RR cells (SupM2) were subjected to size exclusion chromatography. Western blot studies were then performed to identify the distribution of NPM-ALK and FL-NPM1. Most NPM-ALK and FL-NPM1 proteins were found in fractions 1–5, representing the largest protein complexes. In RU cells, a substantial portion was found in fraction 1, in which only a relatively small amount of FL-NPM1 was found. In RR cells, both NPM-ALK and FL-NPM1 appeared to coexist in all 5 fractions. Three independent experiments were performed and results from a representative run are shown.

To provide more direct evidence to support that heterodimers are directly responsible for protecting Myc in RR cells, we performed immunoprecipitation experiments using the nuclear protein extracts of RU and RR cells. Based on our model, we predicted that the heterodimers are much more abundant in RR than RU cells, thereby explaining the differential Myc protein levels between these two cell subsets. This turned out to be the case. As shown in Figure 5A,B, using either anti-ALK or anti-FL-NPM1 for the immunoprecipitation, we found the expression of the heterodimers to be substantially higher in the RR cells. 

Assuming that NPM-ALK proteins rely on the formation of the heterodimers to migrate into the nuclei/nucleoli of ALK+ALCL cells, the relative paucity of the heterodimers in the RU cells suggests that NPM-ALK proteins dissociate from FL-NPM1 after their entry into the nucleus and possibly complex with other proteins. To test this possibility, we performed size exclusion chromatography using nuclear protein lysates extracted from the RU and RR cells, and the results are illustrated in Figure 5C. In the RU cells, NPM-ALK and FL-NPM1 proteins were primarily found in lane 1 and lane 2, which represented the largest protein aggregates. Of note, while most NPM-ALK proteins were found in lane 1, FL-NPM1 was found mostly in lane 2, strongly supporting that a substantial proportion of NPM-ALK proteins in RU cells do not form heterodimers with FL-NPM1. In contrast, both FL-NPM1 and NPM-ALK proteins were found in parallel across all five elution fractions in the RR cells.

### 2.7. The PD-Myc Machinery Sequestered by Nuclear NPM-ALK through the Interaction of Fbw7γ in RR Cells Only

**Figure 6:**
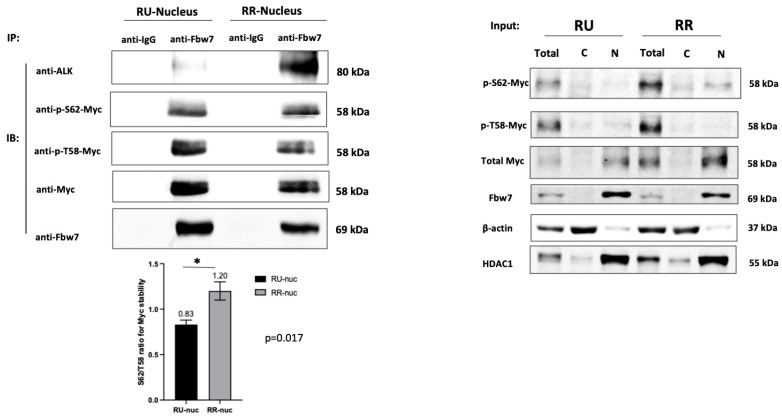
The proteasomal degradation machinery for Myc is relatively inefficient in RR cells, and this finding correlates with its physical association with NPM-ALK. Immunoprecipitation experiments using lysates of RU and RR cells (from SupM2) and antibodies against Fbw7γ showed that the pS62/pT58 ratio was significantly lower in RU cells (0.8 vs. 1.2, * *p* < 0.05). The protein input is shown in the right panel. Three independent experiments were performed and results from a representative study are shown.

Fbw7γ is one of the key components of PD-Myc [41,42]. Normally, it binds to the large complexes of FL-NPM1 and it serves as the docking site for Myc destined for proteasomal degradation [37,43]. Once bound to Fbw7γ, Myc will undergo the sequential phosphorylation and dephosphorylation of two key residues, T58 and S62 [33,41,42]. It has been published that a relatively high phospho-S62/phospho-T58 (pS62/pT58) ratio (i.e., >1.0) correlates with Myc stabilization and a relatively low efficiency of PD-Myc [44]. If our hypothetical model is correct, we expect that a substantial proportion of Fbw7γ in the RR cells binds to the heterodimers and the associated Myc proteins carry a high pS62/pT58 ratio. In contrast, in RU cells, most Fbw7γ binds to large complexes of FL-NPM1 without NPM-ALK and the associated Myc proteins carry a low pS62/pT58 ratio. As shown in Figure 6, our findings are in keeping with our model. For Myc proteins co-immunoprecipitated with Fbw7γ, there was significantly relatively more pT58 than pS62 in the RU cells, with the average pS62/pT58 ratio being 0.8. In contrast, the pS62/pT58 ratio for the RR cells is 1.2.

### 2.8. Colocalization of ALK and NPM1 in the Nuclei of ALK+ALCL Cells Significantly Correlates with a Higher Myc Protein Expression

**Figure 7:**
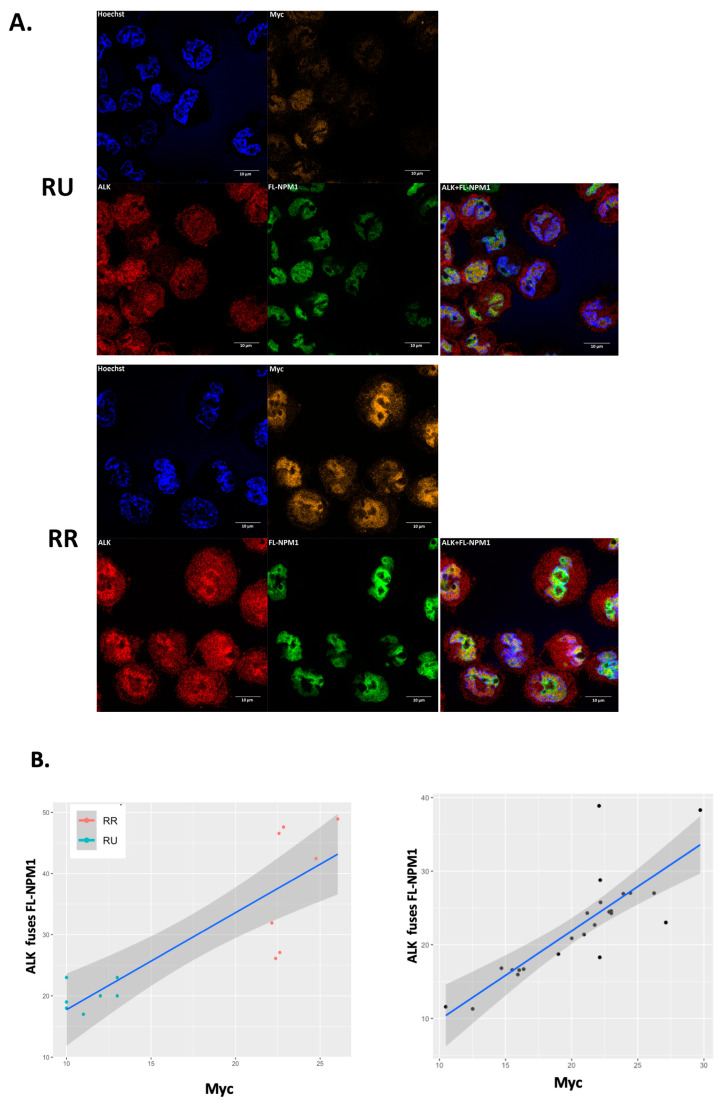
Significantly higher NPM-ALK:FL-NPM1 fusion signals were found in RR cells, and these signals significantly correlate with the Myc signals. (**A**). Representative images of our immunofluorescence staining of RU and RR cells derived from UCONN-L2 cells. Pictures were taken by using a Zeiss LSM710 confocal microscope under a 40X oil lens. Cells were stained with antibodies against conjugated Myc-AF488, ALK with Alexa Fluor 647 as the secondary antibody, and FL-NPM1 with Alexa 555 as the secondary antibody. To show a better correlation between ALK and FL-NPM1, we set the color of Myc-AF488 to orange, while ALK and FL-NPM1 were set to red or green, respectively. A merged ALK and FL-NPM1 channel is shown in the last panel (scale bar (horizontal red line) equates to 10 µm). (**B**). Linear regression analysis and Pearson correlation analysis were performed for RU and RR cells (left panel) or a patient sample (right panel). Seven cells from RU or RR cells, as well as 22 randomly chosen tumor cells, were chosen for analysis, and the pixel intensity calculation was performed by using the ImageJ software. The correlation coefficient is 0.9 for the RU/RR cells and 0.8 for the tumor (*p* < 0.001 and <0.0001, respectively).

To further validate that the heterodimers are more abundant in RR than RU cells, and that the heterodimers are important in protecting Myc from degradation, we performed triple-staining immunofluorescence using these two cell subsets and correlated the fusion signals between ALK and FL-NPM1 (as a marker of the heterodimer) with the Myc signals at a single-cell level. Representative images obtained by using confocal microscopy are shown in Figure 7A. The median pixel intensity of Myc in the RR cells was 22.6 (+/−5.8), which is significantly higher than that of the RU cells (10.0 +/− 2.4) (*p* < 0.0001). The median fusion signal of ALK and FL-NPM1 was 42.4 (+/−10) in the RR cells, which is significantly higher than that of the RU cells with 22.6 (+/−1.5) (*p* < 0.005). When grouping the RU and RR cells together, the Myc signals significantly correlate with the heterodimer signals (Pearson coefficient analysis, r = 0.9, *p* < 0.001) (Figure 7B, left panel). Using a similar method, we studied the ALK+ALCL cells from patient samples at a single-cell level. In a representative case, we analyzed 22 tumor cells, recognized based on their morphology and ALK expression. We found a significant correlation between the signals of Myc and the heterodimers (Pearson coefficient analysis, r = 0.8, *p* < 0.0001) (Figure 7B, right panel).

## 3. Discussion

Myc is a proto-oncogene involved in the regulation of many key biological processes, including cell cycle progression, cell growth regulation, apoptosis, metabolism, and biosynthesis [45]. In embryonic stem cells, Myc plays a key role in the maintenance of pluripotency [46,47,48] and it represents one of the four inducible pluripotent stem cell (iPS) factors [49]. Correlating to its biological importance, Myc is one of the most implicated oncoproteins in human cancer [30]. A high level of Myc expression is frequently identified in cancer cells due to different mechanisms, such as chromosomal translocations involving the locus *8q24* (which houses the *Myc* gene) [50,51]. In the field of ALK+ALCL, the pathogenetic importance of Myc has not been comprehensively studied. Nonetheless, it has been reported that shRNA or pharmacologic inhibition of Myc can lead to a significant decrease in the survival of ALK+ALCL cells in vitro [52]. In a few publications using immunohistochemistry applied to cohorts of ALK+ALCL patient samples, high Myc expression in lymphoma cells was found to significantly correlate with a shorter overall survival [53,54,55]. More recently, our team has found that Myc is a key driver of the CSL phenotype in ALK+ALCL, and the differential expression of Myc proteins in ALK+ALCL cell lines generates intra-tumoral heterogeneity that can be detected as RU or RR cells by using a Sox2 reporter [38,39,40]. 

In this study, we aimed to investigate the mechanism underlying the differential Myc protein levels between RU and RR cells. In view of the importance of Myc in regulating the CSL immunophenotype in ALK+ALCL, we hope to gain insights into how cancer stemness is regulated in these lymphoma cells. In normal cells, Myc is known to have a relatively short half-life of approximately 20 min [56,57]. In this study, we found that the efficiency of PD-Myc is substantially lower in RR cells, resulting in a relatively longer half-life, which correlates to the accumulation of Myc and its higher expression level. Importantly, our data support the model that the nuclear NPM-ALK-FL-NPM1 heterodimers, which exist abundantly in RR but not RU cells, interfere with PD-Myc by sequestering/protecting Myc. The validity of this model is supported by the demonstration of a significant correlation between the abundance of the heterodimers and a high level of Myc in ALK+ALCL cell lines and patient samples. 

Since the discovery of NPM-ALK, the oncogenic functions of NPM-ALK have been attributed almost exclusively to its homodimerization and the resulting autophosphorylation/activation that occur in the cytoplasm of ALK+ALCL cells. The roles of nuclear NPM-ALK, which are believed to exist predominantly in the form of heterodimers, are largely unknown. Our team recently published that the FL-NPM1 component of NPM-ALK binds to FoxM1, an oncogenic transcriptional factor, thereby promoting its DNA binding, transcriptional activity, and oncogenic potential in ALK+ALCL cells [20]. The findings from the current study further support the notion that nuclear NPM-ALK proteins also exert oncogenic effects. In contrast with those of cytoplasmic NPM-ALK, the oncogenic functions of nuclear NPM-ALK appear to be dependent on the versatile binding ability of FL-NPM1 found in the heterodimers. The mechanism by which the NPM-ALK heterodimers interfere with PD-Myc appears to be in parallel with how they regulate FoxM1 by binding to these proteins via FL-NPM1 [20]. 

Our hypothetical model (summarized in Supplemental Figure S2) regarding the role of FL-NPM1 in interfering with PD-Myc stems from the knowledge about the role of NPM1 in the PD-Myc pathway, as reviewed previously [34,35]. In the nuclei of normal cells, NPM1 proteins form large homologous complexes that bind to and stabilize Fbw7γ, a tumor suppressor and E3-ubiquitin ligase. To be recognized by Fbw7γ, Myc proteins need to be phosphorylated first at S62 and then at T58. With the help of phosphatase PP2A, which dephosphorylates Myc at S62 after the phosphorylation of T58, Myc phosphorylated at T58 but not S62 is recognized by Fbw7γ [41,42,58]. Once bound to Fbw7γ that is mostly associated with the large NPM1 complexes, PD-Myc ensues [35]. Accordingly, the pS62/pT58 ratio has been used as an indicator of the efficiency of PD-Myc [44]. Our results have provided at least two pieces of evidence that PD-Myc in RR cells has deviated from this normal pathway: (1) Myc bound to the Fbw7γ protein that is complexed with NPM-ALK carries a high pS62/pT58 ratio, indicating ineffective PD-Myc; and (2) NPM1 proteins are spread out on size exclusion chromatography in RR cells, strongly suggesting that a substantial proportion of NPM1 proteins are not engaged in the formation of homologous polymerization which is required for effective PD-Myc. The relevance of NPM-ALK in this context is supported by the findings that PD-Myc was restored upon the knockdown of ALK or even FL-NPM1. 

If our model is proven to be correct, the fate of the NPM-ALK:FL-NPM1 heterodimers upon their entry into the nuclei of ALK+ALCL cells is very different between RU and RR cells. While the heterodimers will retain their formation in the nuclei of RR cells, NPM-ALK will dissociate from FL-NPM1 in a substantial proportion of the heterodimers. Because nuclear NPM-ALK proteins are known to be mostly unphosphorylated, and they exist as large protein complexes (as shown on size exclusion chromatography), the most likely scenario is that nuclear NPM-ALK proteins in RR cells form large complexes with proteins other than FL-NPM1, and NPM-ALK homodimerization is prevented due to the configuration of these complexes. An understanding of these protein complexes may shed light on how the fate of nuclear NPM-ALK is regulated and, thus, how cancer stemness is regulated in ALK+ALCL cells. In this regard, mass spectrometric analysis of the nuclear NPM-ALK protein complexes in RR cells is underway.

## 4. Materials and Methods

(1) Cell lines and tissue culture

Two ALK + ALCL cell lines, SupM2 and UCONN-L2 (purchased from DSMZ, Braunschweig-Süd, Germany), were used for experiments and cultured in Roswell Park Memorial Institute (RPMI) 1640 media (Gibco, Cat# 11875093, Waltham, MA, USA). Lenti-X 293T cells (purchased from Dharmacon, Cat#HCL4517, Cambridge, UK) were maintained in high-glucose Dulbecco’s modified Eagle’s medium (DMEM, Gibco, Cat# 11965092). All media were supplemented with 10% fetal bovine serum (FBS, Gibco, Cat#16140071) and 1% penicillin and streptomycin (Gibco, Cat# 15140122). All cells were maintained in a 5% CO_2_ atmosphere at 37 °C. RU and RR cells derived from SupM2 and UCONN-L2 were purified with the use of the flow cytometric cell sorter BD FACSAriaTM III (BD Bioscience, Franklin Lakes, NJ, USA). After sorting, cells were always cultured in the presence of 0.5 µg/mL puromycin (Gibco, Cat# A1113803). Details of the generation of RU and RR cells have been described previously [7]. Cells are either commercially available or provided upon request to the corresponding author.

(2) Lipofectamine transfection and lentiviral transduction

For lipofectamine or siRNA transfection, Lipofectamine 2000 or RNAiMax (Addgene, Cat# 11668019 or Cat# 13778075, Watertown, MA, USA) were used, respectively. For lentiviral transduction, Lenti-X 293T cells were first seeded to reach a 60% confluence and then transfected with packaging and transfer vectors Lipofectamine 2000 for 48 h. The detailed viral supernatant collection steps were described before [19]. Cells were washed with cold PBS and resuspended in fresh medium in T25 flasks (Thermo Fisher Scientific, Hillsboro, OR, USA) for incubation 24 h after the second transduction. Short hairpin RNA (shRNA) for NPM1 (Cat# TRCN0000062270) was purchased from Sigma-Aldrich, and short interfering RNA (siGENOME Human ALK siRNA, Cat# M-003103-02-0010) for ALK was purchased from Horizon Discovery (Dharmacon Inc, Boulder, CO, USA). The NPM-ALK (pcDNA3.1) plasmid was a gift from Dr. Stephan Morris.

(3) Antibodies, Western blots, immunoprecipitation, and protein stability assay

Cell lysates were prepared and lysed as described before [19], followed by normal Western blotting steps [19]. All primary antibodies were probed with IRDye 800CW Goat anti-rabbit (Cat# 926-32213) or anti-mouse (Cat# 926-32212) IgG secondary antibody (1:40000, LI-COR Biosciences, Lincoln, NE, USA) with covers to avoid light. The membranes were washed three times with TBS-T and then visualized by the Odessey Western Blot Imager (LI-COR Bioscience). Images were analyzed by the software ImageStudioLite as suggested by LI-COR incorporating the imager instrument (LI-COR Bioscience, Lincoln, NE, USA) to collect band intensity for necessary densitometry analysis. Primary antibodies used as listed: Anti-phospho-ALK (Tyr1604, #3341) and anti-ALK (D5F3, #3633) were purchased from Cell Signalling (Danvers, MA, USA). Anti-c-Myc (Y69, ab32072), anti-T58-pMyc (EPR17923, ab185655), anti-S62-pMyc (EPR17924, ab185656), and anti-Fbxw7 (ab109617) were purchased from Abcam (Cambridge, MA, USA). Anti-B23/Nucleophosmin (NA24, sc-29771) from Santa Cruz Biotechnology (Dallas, TX USA) was used to detect N-terminal NPM1, while anti-B23 (clone FC82291, B0556) from Sigma-Aldrich (St. Louis, MO, USA) was used to detect C-terminal and FL-NPM1. The half-life of Myc was detected by cycloheximide (CHX, ab120093, 10 mg/mL, Abcam, Cambridge, MA, USA) chase assay. Cells were collected every 10 min.

(4) Nuclear/cytoplasmic fractionation and size exclusion chromatography

Collected cells were washed with cold PBS and then fractionated into cytoplasmic and nuclear fractions by the Pierce NE-PER kit (Cat# 78833, Fisher Scientific, Hillsboro, OR, USA) according to the manufacturer’s instructions. The nuclear fractions then went through a 0.45 μm filter to get rid of large particles before being sent for size exclusion chromatography (SEC). SEC was performed through the High-Performance Liquid Chromatography service provided by the Lipidomics Core Facility at the University of Alberta. Collected fractions were utilized for further Western blotting.

(5) Immunofluorescence staining

Anti-Myc (Y69) conjugated with Alexa Fluor 488 antibody (ab190026, Abcam, Cambridge, MA, USA) was used (1:200 dilution) in the immunofluorescence assay. Antibodies against ALK (1:200 dilution) and C-NPM1 (1:200 dilution) were used in immunofluorescence double staining. The procedures for the immunofluorescence assay were briefly described below. Cells were first placed on a coverslip sitting in a 12-well plate with PBS for at least 30 min, and then fixed with 10% formaldehyde for 10 min, followed by a 5 min washing step using PBS. Cells were then permeabilized using 0.5% Triton X-100 in PBS for 10 min, followed by a 5 min washing step using PBS. Primary antibodies diluted in PBS were then added to cells to incubate overnight at 4 °C on a shaker. For formalin-fixed, paraffin-embedded tissue sections, slides were deparaffinized and hydrated. Heat-induced epitope retrieval was performed using EDTA buffer (pH = 9, E1161-1000ML) and a pressure cooker using the microwave. Tissue sections were then permeabilized for 15 min with 0.25% Triton X-100 (Cat# X100-100ML, Sigma-Aldrich, St. Louis, MO, USA) in 1× PBS, followed by the block with 1× PBS containing 1% BSA (Cat# A3294-100G, Sigma-Aldrich, St. Louis, MO, USA) for 30 min at room temperature. The tissue sections were incubated with primary antibodies against ALK and C-NPM1 which are diluted in 1× PBS overnight at 4 °C. The next day, after three times of washes with 1× PBS (10 min each), tissue sections were incubated with secondary antibodies (Alexa Fluor 594 goat anti-rabbit antibody, Cat# A-11037, and Alexa Fluor 647 goat anti-mouse antibody, Cat# A-21236, Invitrogen, Burlington, CA, USA), diluted in 1× PBS, 1:200 for 1 h at room temperature, followed by washing three times with 1× PBS (10 min each). The slides were then followed by a blocking process with 5% normal rabbit or mouse serum in PBS followed by another three rounds of washing steps. Conjugated Myc-AF488 antibody was then added to the slides for overnight incubation at 4 °C. After washing in 1× PBS, tissues were incubated in Hoechst 33342 (Sigma-Aldrich, B2261, 1:5000 dilution, St. Louis, MO, USA) for 10 min, followed by washes in 1× PBS and mounted with Permount Mounting Medium (Fisher Scientific, #SP15-100, Hillsboro, OR, USA). Cells were visualized with a Zeiss LSM510 confocal microscope (Carl Zeiss, Heidelberg, Germany) using a 40X oil lens at the Core Cell Imaging Facility, Cross Cancer Institute, University of Alberta, Edmonton, Canada.

(6) Image processing and statistical analysis

The intensity of fluorescence pixels was calculated by ImageJ software (NIH and Laboratory for Optical and Computational Instrumentation, LOCI, University of Wisconsin, Madison, WI, USA). The following statistical analyses were performed by Rstudio. To confirm the high Myc expression level correlates to NPM-ALK:FL-NPM1 heterodimer, the data were first examined to fit normal distribution with potential positive correlation based on the scatterplot, Q-Q plot, and Shapiro–Wilk analysis (data not shown). Pearson correlation analysis was then applied to confirm the relationship between Myc pixel numbers and the colocalized ALK and NPM1 pixels.

## 5. Conclusions

To conclude, we have presented a hypothetical model by which the differential Myc expression between RU and RR cells is generated. Our model focuses on the importance of the NPM-ALK:FL-NPM1 heterodimers, which persist mostly in RR but not RU cells. We have presented evidence that the heterodimers directly interfere with PD-Myc by disrupting the normal interactions among Fbw7γ, NPM1, and Myc. In light of the importance of Myc protein levels and cancer stemness, further investigations into these aberrancies in cancer cells might help identify new biomarkers for CSL cells and novel therapeutic targets.

## Data Availability

The datasets used and/or analyzed during the current study are available from the corresponding author upon reasonable request.

## Supplementary Materials

The following supporting information can be downloaded at: https://www.mdpi.com/article/10.3390/ijms241814337/s1.
