# Supplementary figures and images for: Nuclear NPM-ALK Protects Myc from Proteasomal Degradation and Contributes to Its High Expression in Cancer Stem-Like Cells in ALK-Positive Anaplastic Large Cell Lymphoma
Source: Int J Mol Sci. 2023 Sep 20;24(18):14337. doi: 10.3390/ijms241814337 (PMC10531997; doi:10.3390/ijms241814337)

**A.****HEK293**

|                       |   |   |   |   |
|-----------------------|---|---|---|---|
| <b>MG132</b>          | - | + | - | + |
| <b><i>NPM-ALK</i></b> | - | - | + | + |

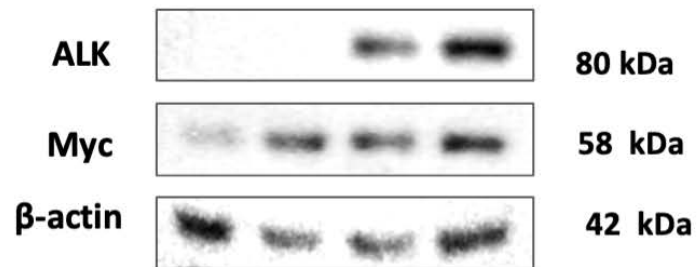

Lane 1 Lane 2 Lane 3 Lane 4

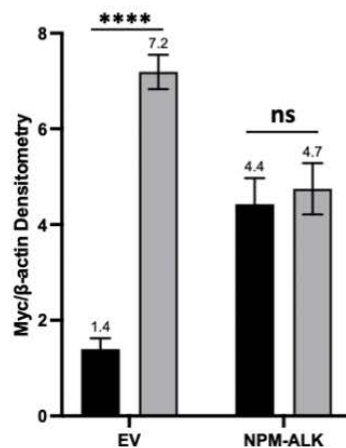**B.****HEK293**

|                       |   |   |   |   |
|-----------------------|---|---|---|---|
| <b><i>NPM-ALK</i></b> | - | - | + | + |
| <b>shNPM1</b>         | - | + | - | + |

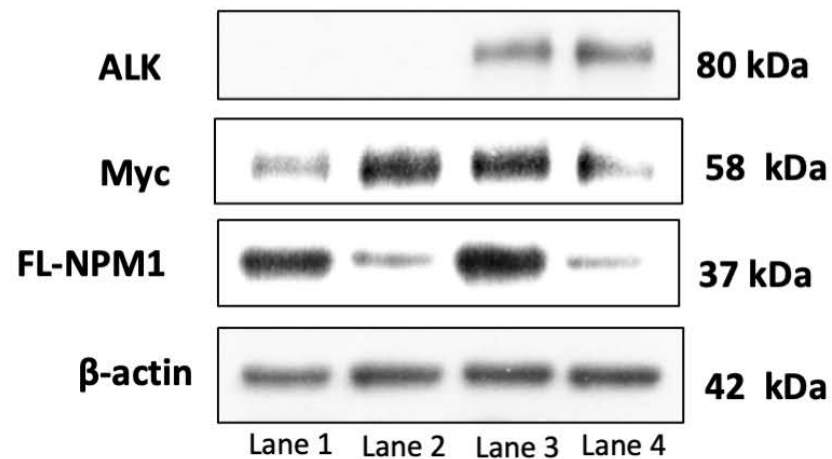

Lane 1 Lane 2 Lane 3 Lane 4

Supplement: Supplementary file 1 [file ijms-24-14337-s001.zip › Figures in slides-revised/Figure 1.pdf]

**A.****SupM2****RU****RR****MG132**    -    +    -    +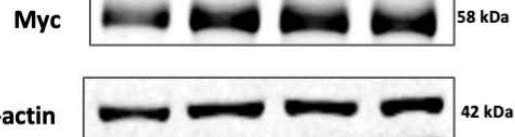

Lane 1   Lane 2   Lane 3   Lane 4

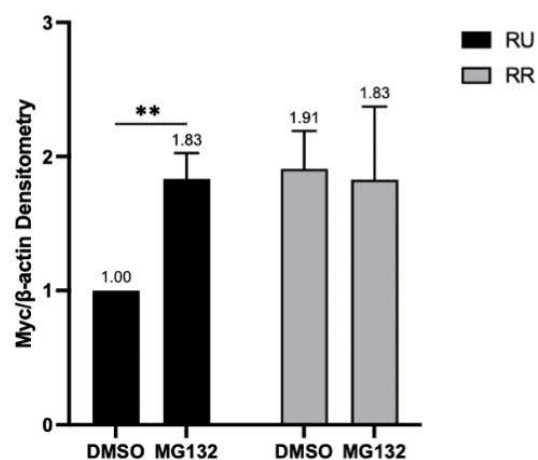**B.****SupM2****RU****RR**

+CHX (min)    0    10    20    30    40    50    60    0    10    20    30    40    50    60

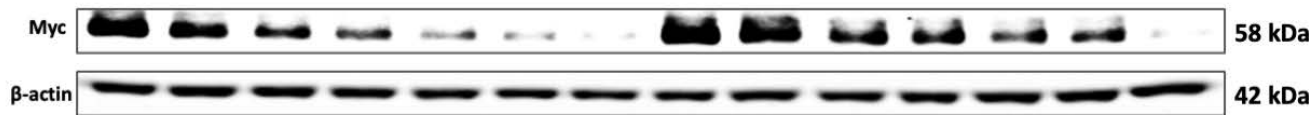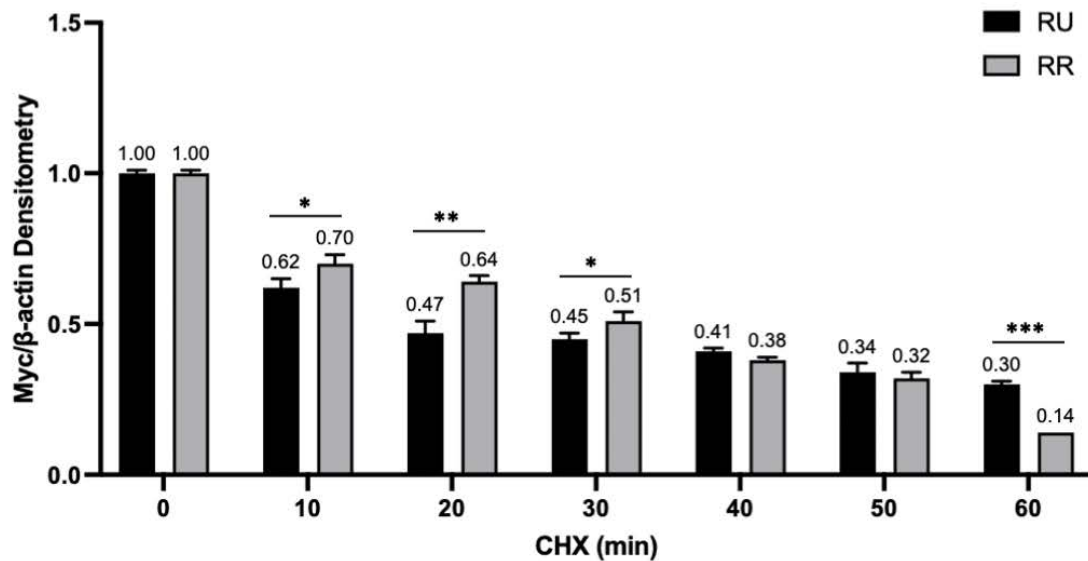

Supplement: Supplementary file 1 [file ijms-24-14337-s001.zip › Figures in slides-revised/Figure 2.pdf]

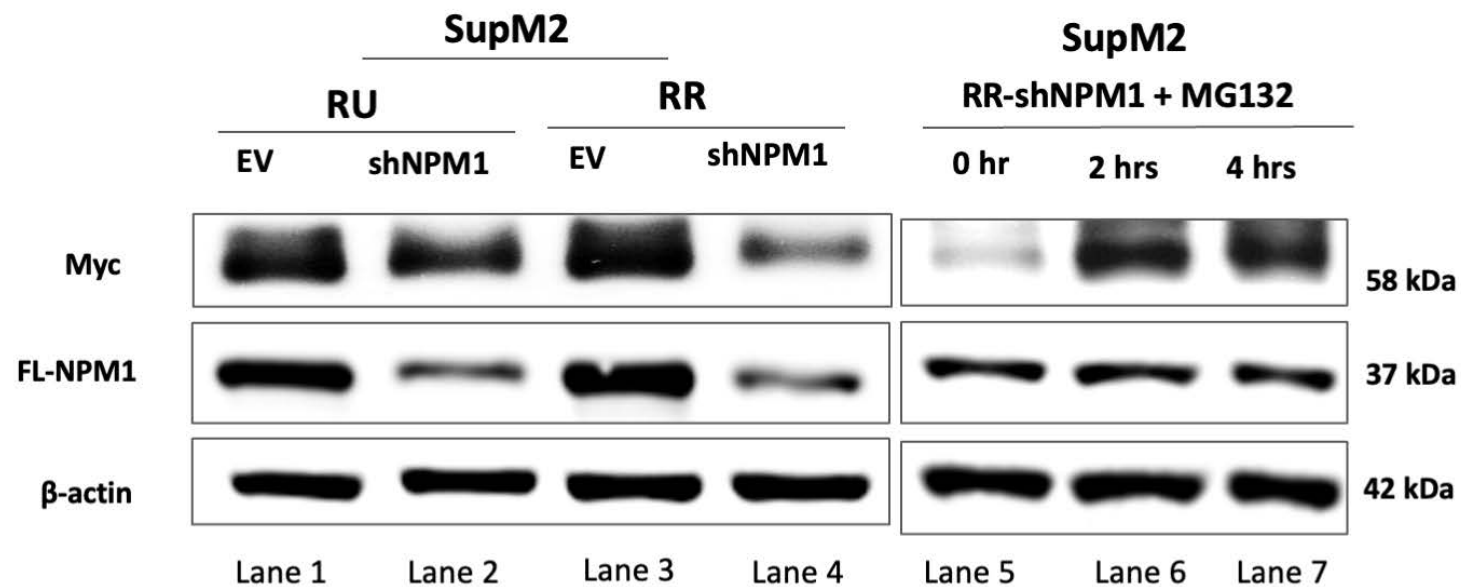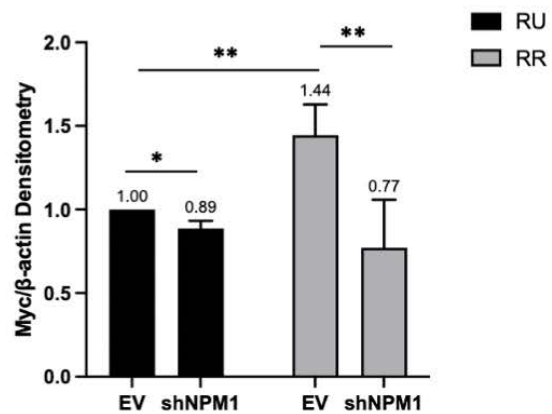

Supplement: Supplementary file 1 [file ijms-24-14337-s001.zip › Figures in slides-revised/Figure 3.pdf]

A.

SupM2 RR

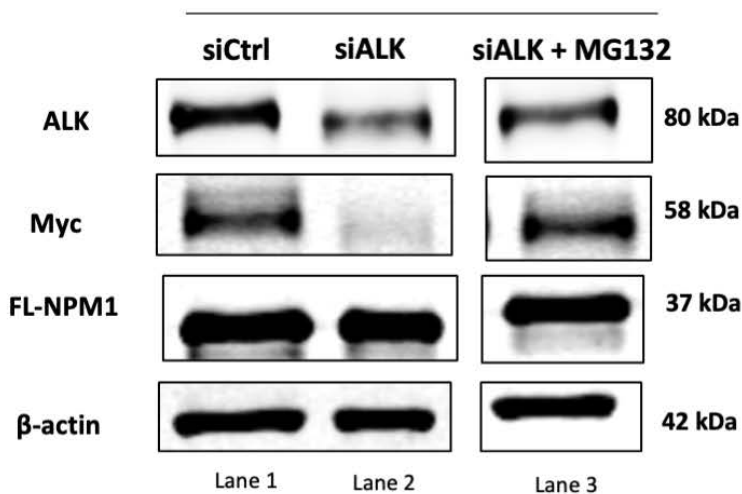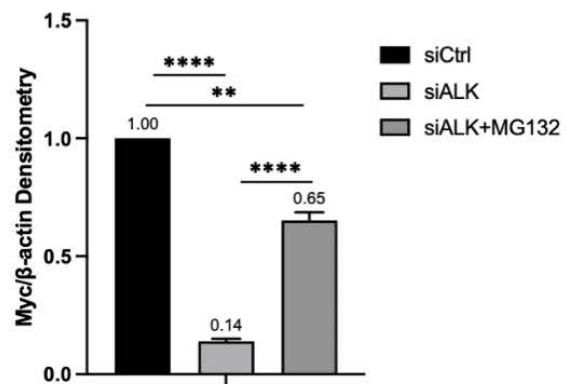

B.

SupM2 RR

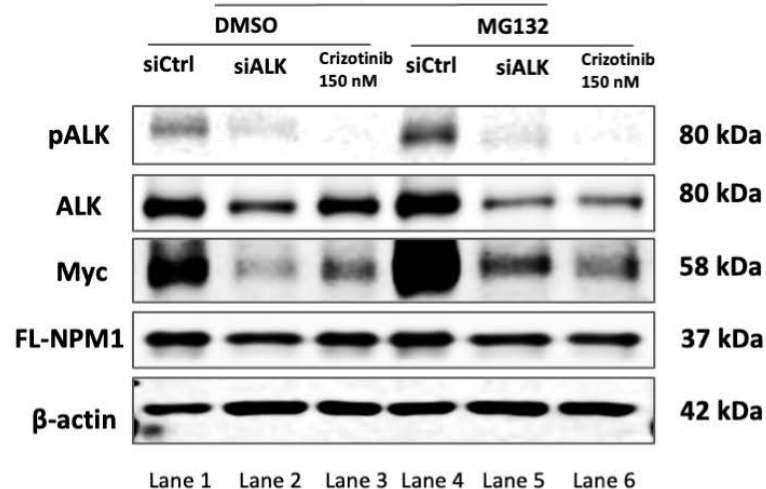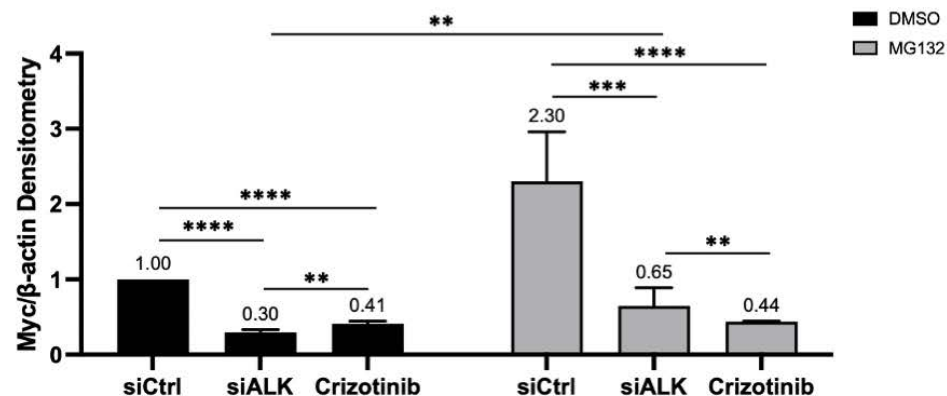

Supplement: Supplementary file 1 [file ijms-24-14337-s001.zip › Figures in slides-revised/Figure 4.pdf]

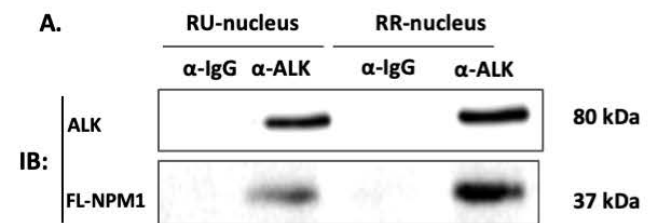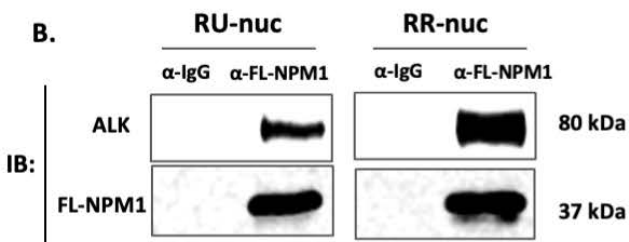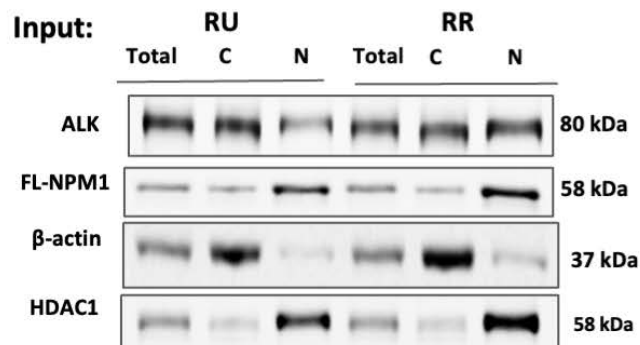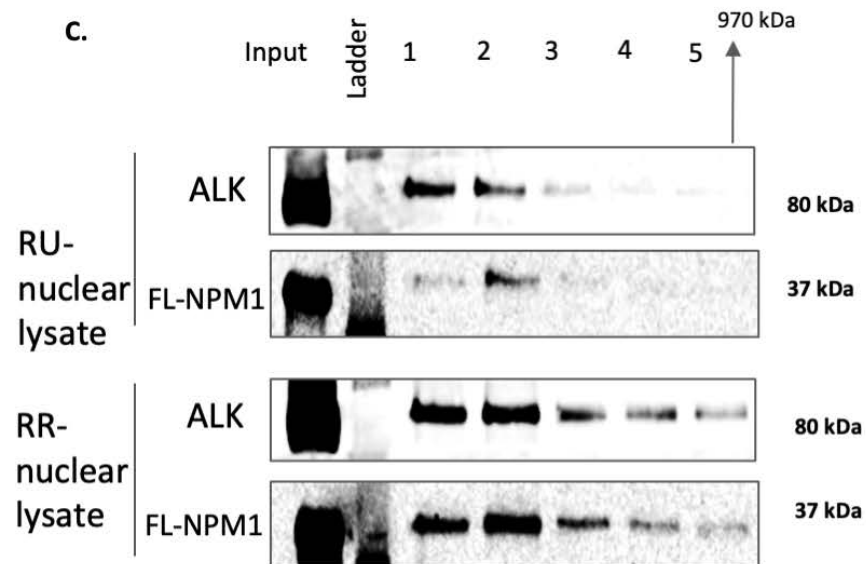

Supplement: Supplementary file 1 [file ijms-24-14337-s001.zip › Figures in slides-revised/Figure 5.pdf]

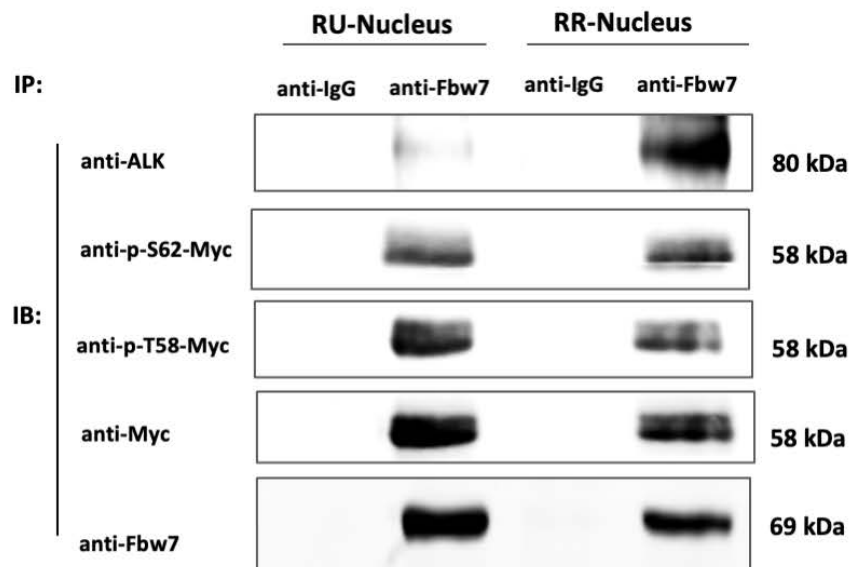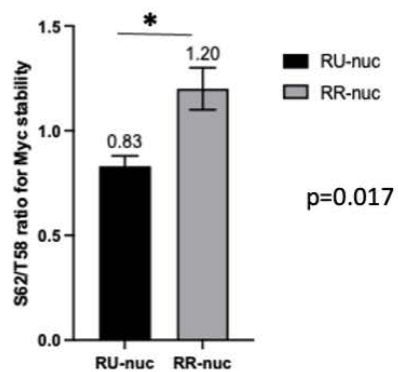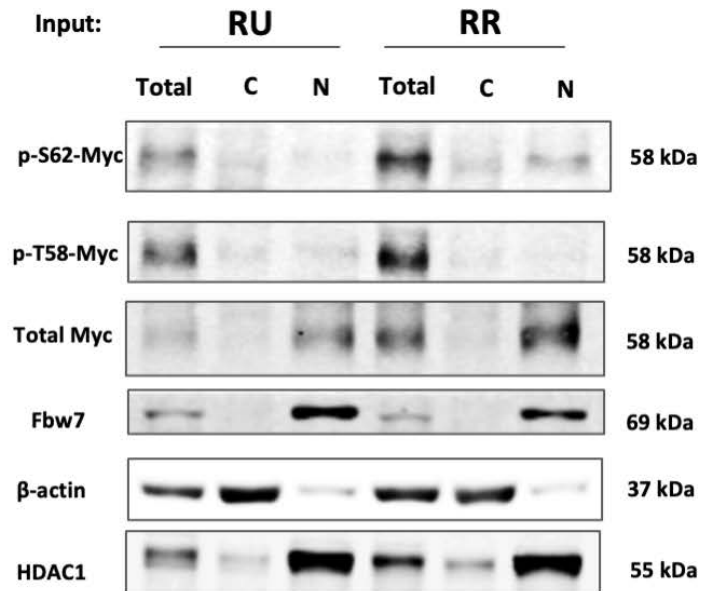

Supplement: Supplementary file 1 [file ijms-24-14337-s001.zip › Figures in slides-revised/Figure 6.pdf]

**A.**

**Hoechst**

**Myc**

**ALK**

**FL-NPM1**

**ALK+FL-NPM1**

**RU**

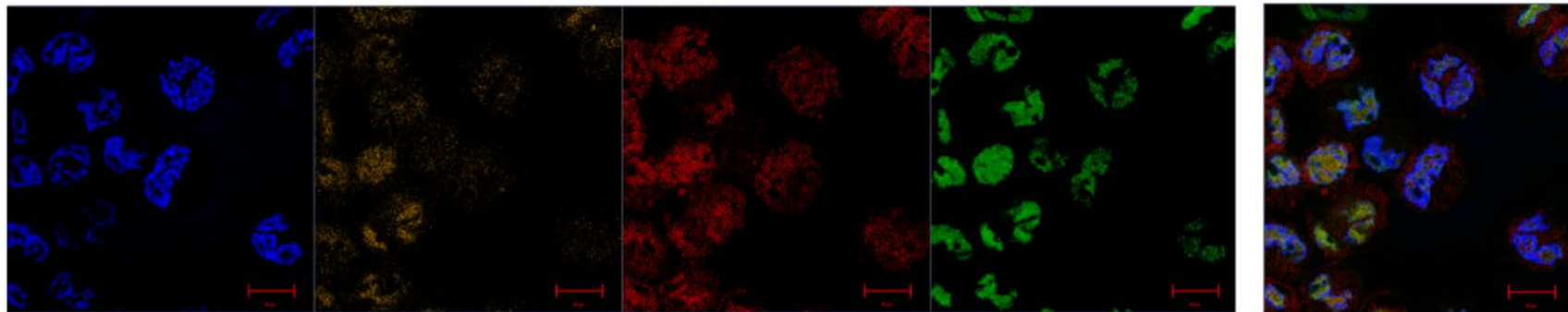

**RR**

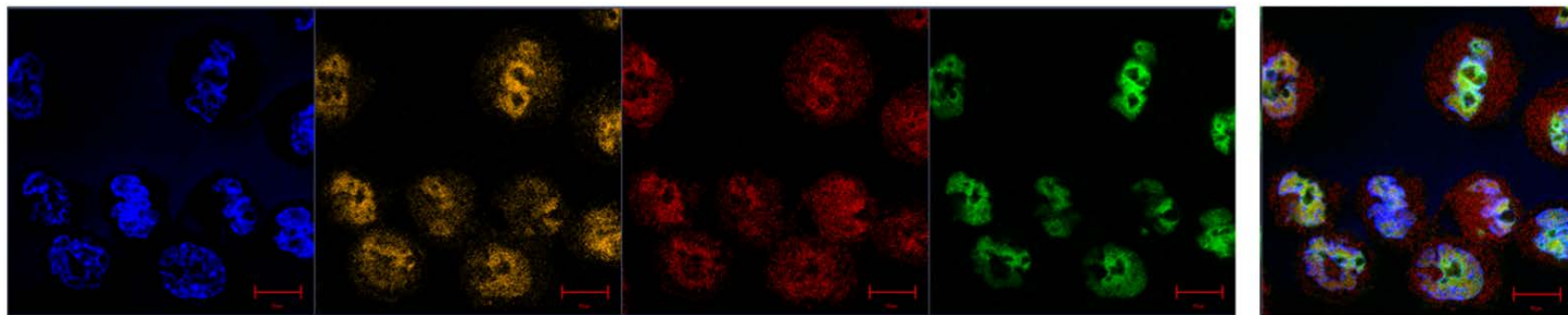

Supplement: Supplementary file 1 [file ijms-24-14337-s001.zip › Figures in slides-revised/Figure 7A.pdf]

**B.**

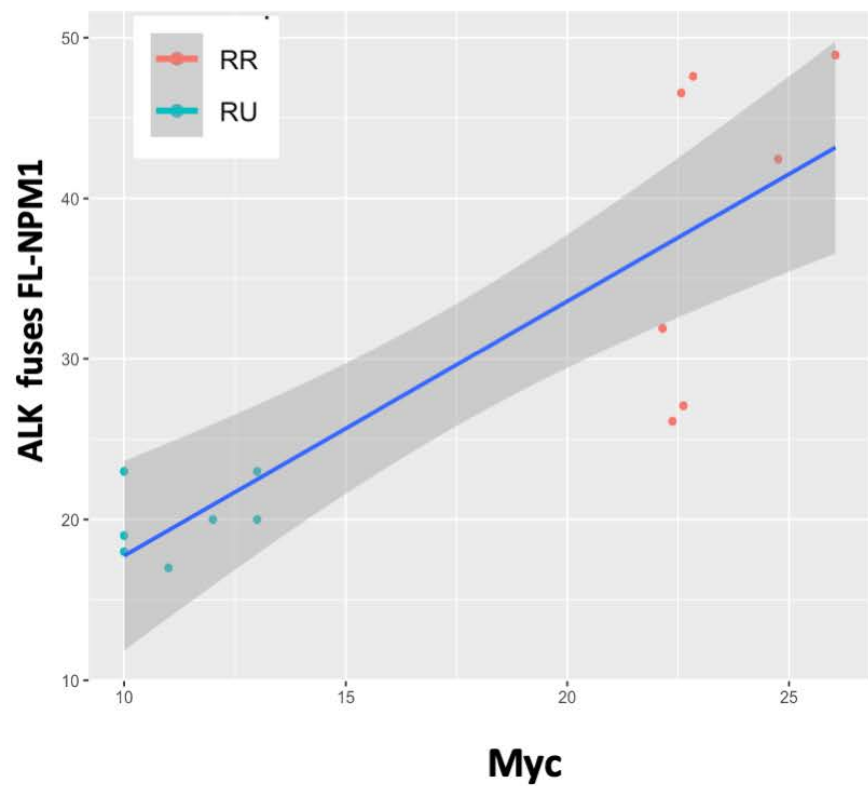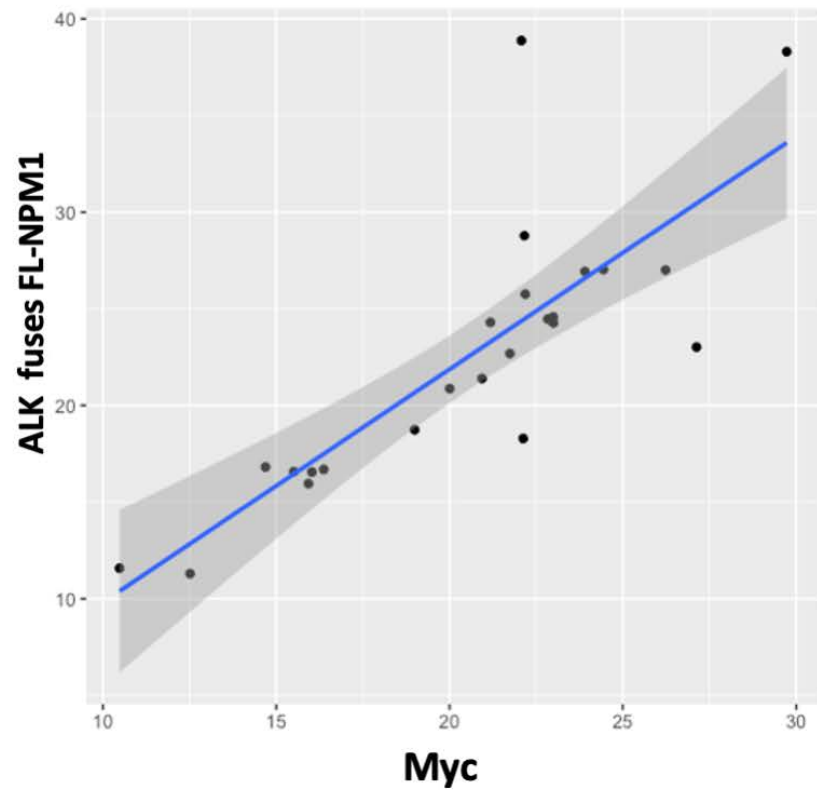

Supplement: Supplementary file 1 [file ijms-24-14337-s001.zip › Figures in slides-revised/Figure 7B.pdf]

Hoechst

Myc

ALK

Merged

EV

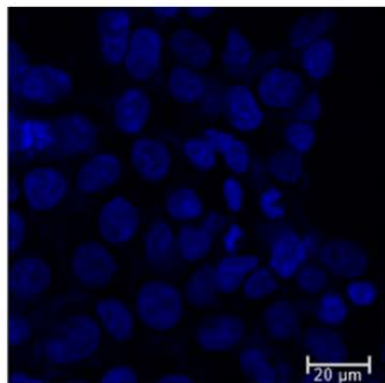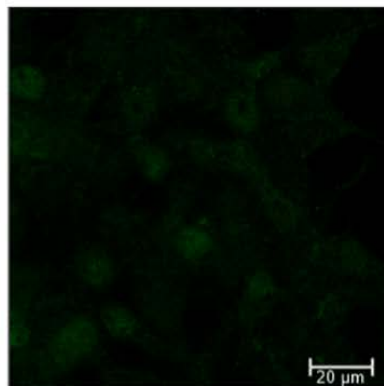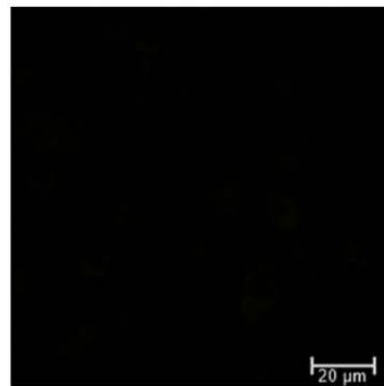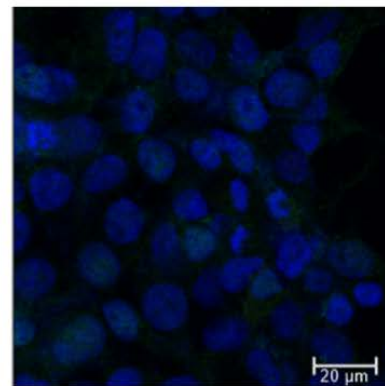

NPM-ALK

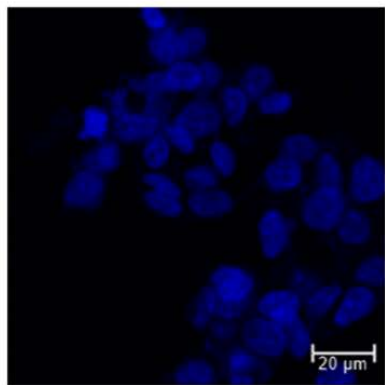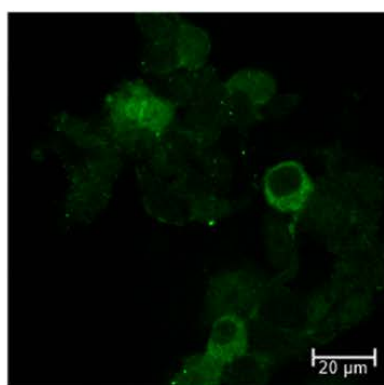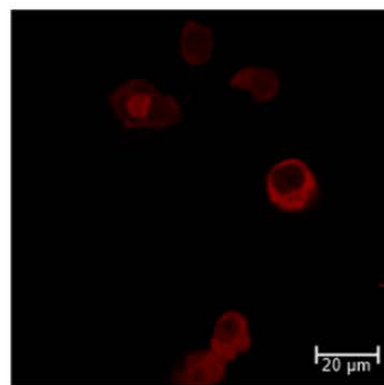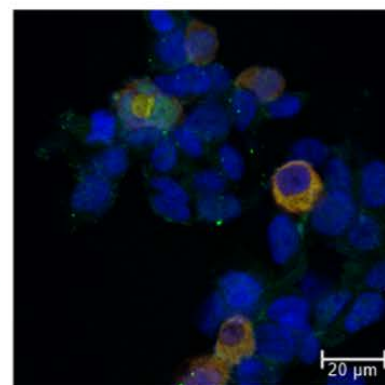

Supplement: Supplementary file 1 [file ijms-24-14337-s001.zip › Figures in slides-revised/Supplemental Figure S1.pdf]

## Bulk (RU) Cells

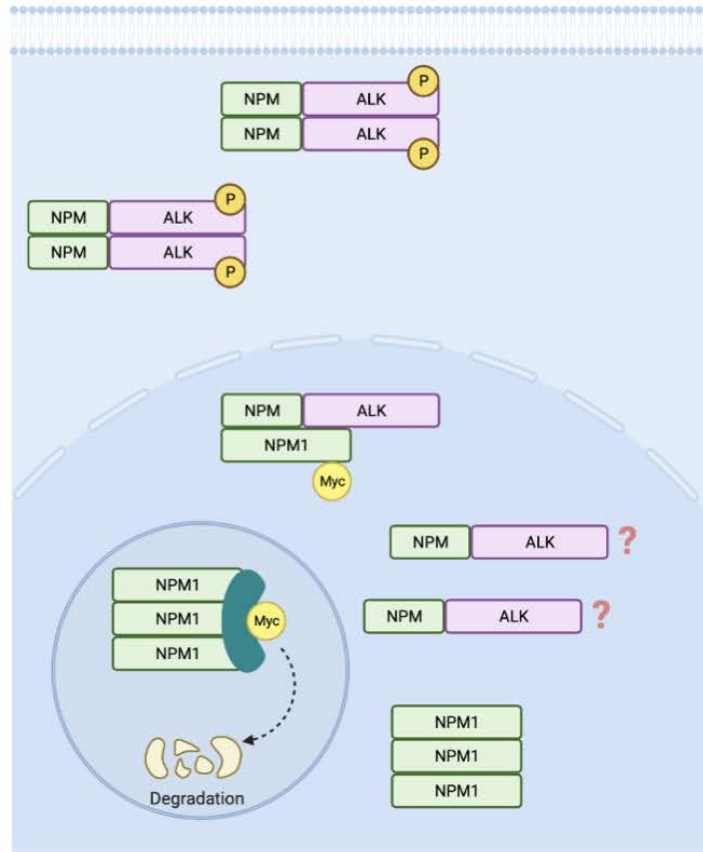

## Stem-Like (RR) Cells

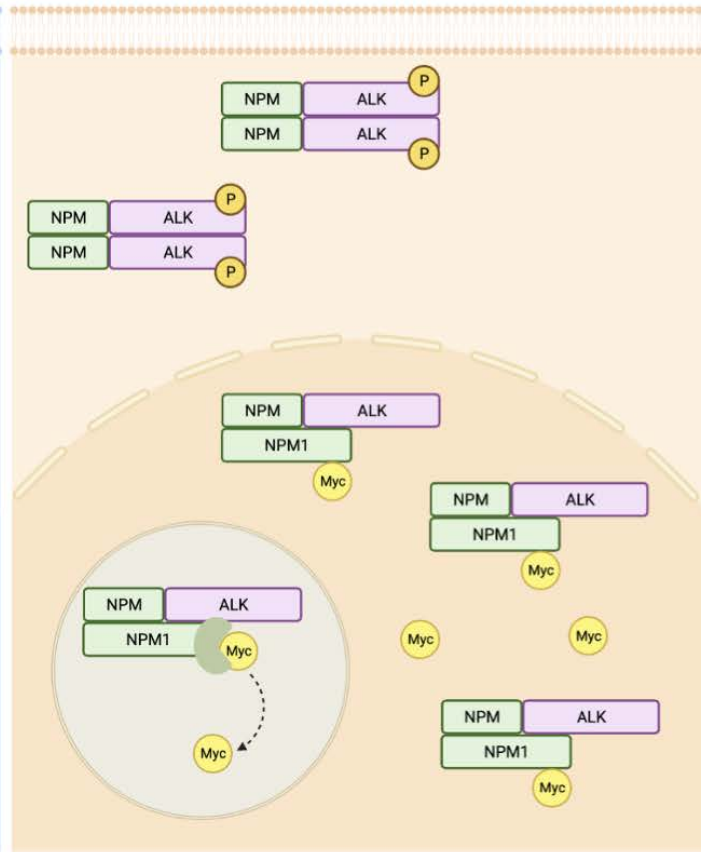

Supplement: Supplementary file 1 [file ijms-24-14337-s001.zip › Figures in slides-revised/Supplemental Figure S2.pdf]
